# The Multi‐Author Practical Scoping (MAPS) Model: Practical Guidance for Collaborative Scoping Reviews in Health Professions Education

**DOI:** 10.1111/tct.70523

**Published:** 2026-09-14

**Authors:** Heidi Stelling, Megan Brown, Alex Boyle, Christina Higgins, Bryan Burford, Gillian Vance

**Affiliations:** ^1^ Newcastle University Newcastle upon Tyne UK

**Keywords:** guide, methodology, practical, scoping review, team

## 1 Introduction

Scoping reviews are widely used to explore complex or emerging topics in health professions education (HPE), but their growing popularity has been accompanied by concerns about methodological consistency and the practical challenges of conducting high‐quality reviews [1]. Existing frameworks—most notably Arksey and O'Malley's model [2] and its subsequent refinements [3], along with Joanna Briggs Institute guidance [4]—offer strong conceptual foundations but limited practical direction on how mixed‐experience teams can apply these steps collaboratively.

Mixed‐experience collaborative approaches offer important educational and scholarly benefits but despite their widespread use in HPE practical guidance translating methodological principles into step‐by‐step processes remains limited. Recent guidance by Church and Sandars [5] provides a clear and practical introduction to the purpose, conduct and reporting of scoping reviews in HPE. The MAPS (Multi‐Author Practical Scoping) model builds on this methodological foundation but addresses a different practical problem: how mixed‐experience, multiauthor teams can organise and undertake the work of a scoping review collaboratively. This is particularly important where scoping reviews are used not only as evidence synthesis methods but also as opportunities for research training and capacity building. In these contexts, teams require more than knowledge of review stages; they also need structured processes for distributing work, maintaining consistency, supporting novice reviewers, documenting decisions and enabling meaningful collaborative interpretation.

Created and refined by supervising collaborative scoping reviews across three cohorts of interns in Newcastle University's Co‐Production initiative [6], MAPS provides a structured, replicable process for team‐based scoping reviews. By providing an operational framework for mixed‐experience review teams, MAPS aims to support methodological rigour, facilitate research capacity building and enable meaningful collaboration between students, early‐career researchers and experienced academics. MAPS' comprehensive stepwise approach guides teams through procedural tasks, making efficient use of limited supervisor time and availability—well recognised barriers to engaging undergraduates in research activity [7, 8]. Research‐active institutions strengthen evidence‐based practice with undergraduate training clearly identified as a key mechanism for achieving these benefits [9]. However, despite the GMC's mandate [10] to develop undergraduate research competence in the United Kingdom and strong student demand for more opportunities [11], medical schools worldwide continue to provide insufficient formal research training (ibid).

This Toolbox presents the MAPS Model as a practical guide for conducting collaborative, high‐quality scoping reviews in HPE. MAPS does not replace the methodological stages described by Arksey and O'Malley [2]; rather, it specifies how roles, shared tools, calibration, supervision and collaborative interpretation can be practically organised when reviewers have different levels of experience. The sections below therefore describe both how work is undertaken at each stage and how interaction between Lead and Junior Reviewers supports feedback, reflexivity and the development of research capability.

*It specifies how roles, shared tools, calibration, supervision and collaborative interpretation can be practically organised when reviewers have different levels of experience*.

## 2 The MAPS Model

**FIGURE 1:**
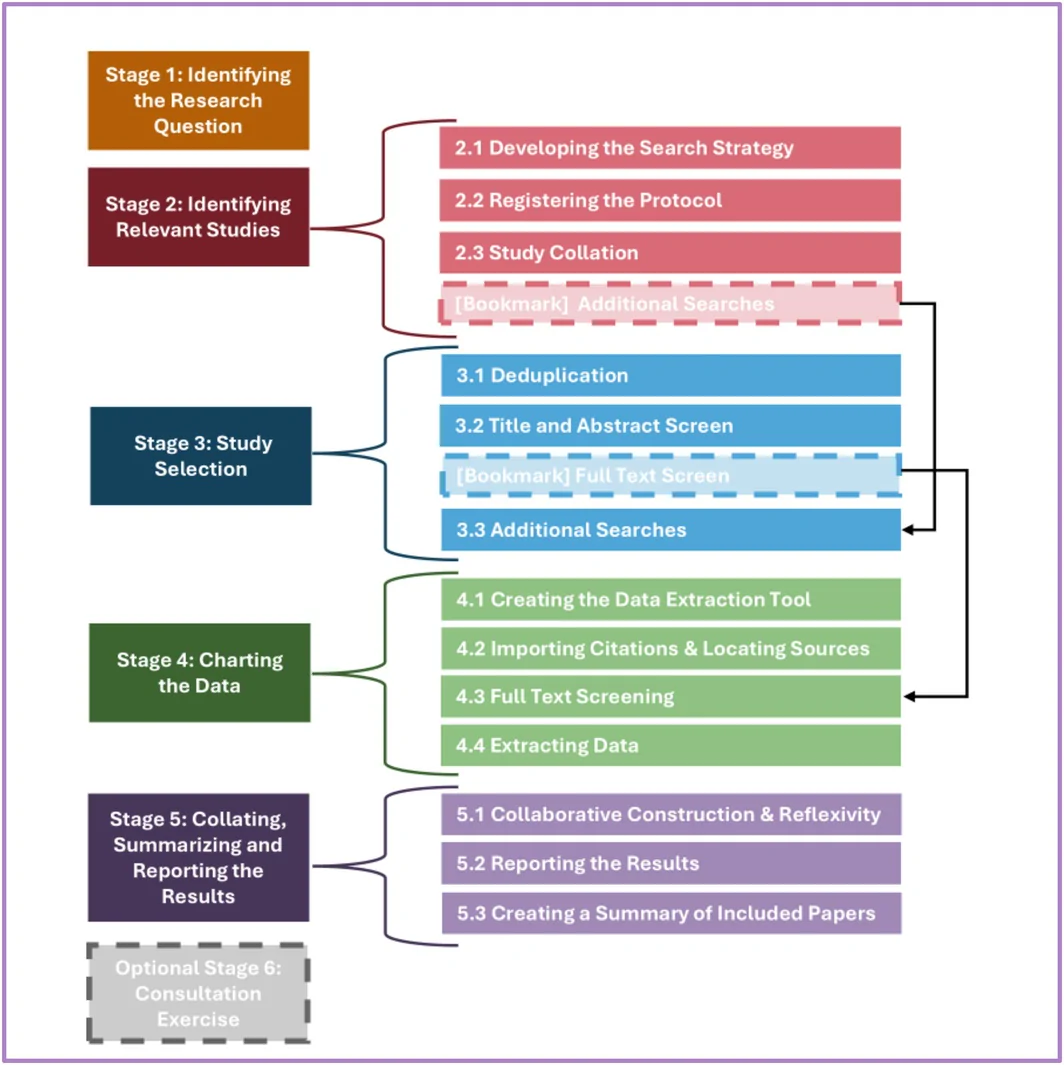
The MAPS model overview.

The MAPS model (Figure 1) aligns with Arksey and O'Malley's framework, with ‘bookmarks’ indicating the placement of original stages, and arrows illustrating where our team found each step most practical to undertake. In our implementation, undergraduates (Junior Reviewers) undertook most tasks, whereas an early‐career researcher (lead reviewer) coordinated the process, supported by a more experienced colleague. This role structure supported capacity building throughout the career continuum.

*This role structure supported capacity building throughout the career continuum*.

### 2.1 Stage 1: Identifying the Research Question

Consistent with Arksey and O'Malley's framework, this stage engages key stakeholders and conducts preliminary searches to refine the study's rationale and ensure its relevance to practice and policy. MAPS adds a structured role for Junior Reviewers within this early work. With guidance from the lead reviewer, participation in preliminary searching and stakeholder discussion helps less‐experienced reviewers learn how the scope, feasibility, and relevance of a review question are negotiated rather than receiving a fully formed question.

### 2.2 Stage 2: Identifying Relevant Studies

MAPS distributes this stage across junior reviewers, the lead reviewer and an information specialist. Junior reviewers undertake the initial formulation and documentation of the search, whereas feedback and collaborative protocol development make the lead reviewer's methodological reasoning governing key research choices visible, critical for methodological skill development among novice researchers.

#### 2.2.1 Developing the Search Strategy

Research questions are translated into searchable terms to produce a clear, reproducible search strategy. This is collaboratively drafted by junior reviewers and refined by the lead reviewer alongside an information specialist, whose input is essential for refining terms and selecting databases. The search is then run in the agreed databases, and the strategy is saved in a shared folder for later reporting.

#### 2.2.2 Registering the Protocol

We recommend registering the review protocol after finalising the search strategy but before running the searches. We used the Open Science Framework (OSF), although other established research repositories (e.g., Zenodo or Figshare) may also be suitable. Download the registration template into a shared document so the team can draft collaboratively; dividing sections between members encourages broad input while maintaining efficiency.

*Download the registration template into a shared document so the team can draft collaboratively; dividing sections between members encourages broad input while maintaining efficiency*.

#### 2.2.3 Study Collation

All records from completed searches are collated before screening begins. Each database output must be exported and saved in formats compatible with your chosen software (see Table 2) (e.g., CSV for Microsoft Excel, RIS for Rayyan and RefMan for Zotero). Keep all original exports untouched as a backup in the shared folder. The lead reviewer creates a new project in your chosen systematic review screening platform (see Table 2) and imports the compatible files from each database. Most review screening platforms preserve source metadata so using clear file names supports easy tracking and verification later in the process (Top Tip 1).

**Table:** 

Top tip 1: PRISMA data
Keep a running record of PRISMA [12] numbers throughout the screening process. Document not only total numbers at each stage but also breakdowns by database, as this level of detail can be difficult to reconstruct later and strengthens the transparency of the final review.

### 2.3 Stage 3: Study Selection

MAPS treats piloting, double‐screening and discussion of conflicts as both a quality‐assurance and a learning process. The steps described below support junior reviewers to explain and compare decisions, receive feedback and refine their application of the eligibility criteria before undertaking the remaining screening more independently.

#### 2.3.1 Deduplication

If available, initial deduplication can be performed using the screening platform's automated function and manual checked for accuracy by the lead reviewer. The deduplicated records are then divided among screening team members, and the ‘blind’ function is enabled to maintain independent screening. The team revisit protocol eligibility criteria and agree exclusion labels. While recording reasons for exclusion is not mandatory at the title and abstract stage, doing so scaffolds learning for less experienced reviewers and allows easy filtering and review of uncertain records.

#### 2.3.2 Title and Abstract Screen

This step is divided into pilot screening and full screening, reflecting MAPS' scaffolded approach for less experienced reviewers.

##### 2.3.2.1 Pilot Screening

For the pilot, take a sample of 100 records from the set assigned to each reviewer. Each reviewer screens their initial 100 records; 10% of each reviewer's pilot set is then double‐screened for consistency. Pilot samples and subsamples should be clearly and systematically labelled (see Top Tip 2). Following the pilot, a team meeting is held to (1) review and refine eligibility criteria if needed and (2) resolve double‐screening conflicts.

##### 2.3.2.2 Full Screening

Once confident in the criteria, reviewers screen remaining records with a random 10% sample double‐screened for quality assurance. Consistent labelling is maintained throughout, and regular meetings support conflict resolution, shared learning and screening consistency. Disagreements should first be discussed between the reviewers with reference to the eligibility criteria. Where uncertainty remains, we recommend inclusion at this stage, allowing decisions to be revisited during full‐text screening where additional contextual information is available.

#### 2.3.3 Additional Searches

Technically part of ‘Stage 2: Identifying Relevant Studies’, additional searches often occur after database screening to address gaps and expand the citation pool. MAPS incorporates sources from (1) grey literature, (2) targeted academic searches and (3) backwards and forwards citation tracking using a standardised spreadsheet template. A reusable example of this template is provided in Supporting Information S1 (Tables S4 and S5) to support adaptation by other review teams. Individual spreadsheets are used to maintain an audit trail in the searching process, but any new sources are systematically labelled, added to the ‘Extras’ tab (Supporting Information S1, Table S2) of the main spreadsheet for data extraction and imported to your selected reference manager software. Google and Google Scholar were predominantly used for additional searches, and screening followed a ‘100‐plus‐10’ approach (a pragmatic method of screening a further 10 sources if there was a potential inclusion in the preceding 10). Author contact, to identify additional or unpublished material, was optional but followed standardised methods across the team.

**Table:** 

Top Tip 2: Consistent labelling
Use a simple, standardised labelling system from the outset to ensure traceability across all stages. Key principles: Choose a fixed number format (e.g., two‐ or three‐digit numbering [007 rather than 7]) to keep records sortable. Label files and batches consistently by including the key elements (e.g., database/source, stage, reviewer name and record count). Apply the same structure to pilot samples, double‐screened subsets and additional searches so files can be identified at a glance. Do not rename original files—create new labels only when exporting or generating subsets and keep a record of all changes. A clear, consistent system prevents confusion, supports auditability and becomes invaluable when working with multiple reviewers and large datasets.

### 2.4 Stage 4: Charting the Data

MAPS divides charting into tool development, familiarisation, piloting and full extraction. This staged approach helps junior reviewers understand how research questions are translated into extractable data and allows interpretations to be tested and refined with the team before full extraction begins.

*This staged approach helps junior reviewers understand how research questions are translated into extractable data and allows interpretations to be tested and refined with the team before full extraction begins*.

#### 2.4.1 Creating the Data Extraction Tool

Preliminary extraction decisions, outlined in the protocol, should be revisited as early reading often reveals additional fields. Extraction should be focussed on the research questions to maintain efficiency. Core extraction fields will be project‐specific, but the extraction tool should be designed to support collaboration and guide novice researchers. Although not required within a scoping review, we included an optional quality column for relevant observations, which reviewers found useful. Once agreed, a team member designs the extraction tool (see Tables 1 and S1), which is then duplicated into separate tabs for each reviewer, plus an ‘Extras’ tab (Supporting Information S1, Table S2) for sources from additional searches (see Section 2.3.3). Column headings must not be changed without full team agreement, as all tabs must remain aligned.

**TABLE 1:** Suggested columns layout in data extraction tool.

Section	Heading	Notes
	Source number	See advice in Stage 4.2 on systematic numbering
Meta‐data	Author, title, year …	Standard bibliographic details for each source.
Full text screen	Paper sourced^a^	[Yes/no/interlibrary loan request/scanned copy request] If not directly available through institutional/open access record how the full text was obtained in the notes section.
	Decision^a^	[Include/exclude/undecided] Keeping an undecided option supports less experienced reviewers, creates a ‘safety net’ and helps generate productive team discussion.
	Exclusion category^a^	[Content, publication type, language, other, NA] Categories will depend on the eligibility criteria. Use NA for included papers.
	Exclusion reason	Free text field to provide more detailed justification. Useful where reviewers are unsure how to categorise an exclusion.
	Reviewer	Initials of the primary reviewer.
	Checker [1]	Initials of the second reviewer for the 10% double‐screening sample. The actual screening occurs in a separate spreadsheet. Conflicts are discussed in team meetings.
Data extraction	Study design, results, conclusions, recommendations …	Columns should be tailored to the project's specific research questions. Focus extraction on what is necessary to answer these questions. Regular team meetings safeguards consistency and allow for amendments if needed.
Notes	Quality, relevance to the review, questions …	Optional but useful space for reviewers to record uncertainties or observations for later discussion. See main text for further discussion on quality considerations.
Admin	Extractor	Initials of the person completing data extraction.
	Checker [2]	Initials of the second extractor for the 10% double‐extraction sample. The actual double‐extraction occurs in a separate spreadsheet Findings from this process were discussed in weekly meetings.
	Suitable for discussion^a^	[Yes/no] Papers excluded at full text can also be flagged here if they provide useful background material.
	Reason for discussion	Free text—captures why a paper may merit discussion despite not meeting eligibility criteria.
	Complete^a^	[Yes/no] Optional checkbox or status column. Tracking completion provides an overview of progress and can be reassuring during lengthy stages.

*Note:* Suggested options are provided in square brackets.

^a^ Where a drop‐down menu may be useful.

#### 2.4.2 Importing Citations and Locating Sources

Records are exported from the screening platform for full‐text screening in a format compatible with the selected spreadsheet and data management software and the references manager (e.g., CSV for Microsoft Excel and RefMan for Zotero). Before exporting any data, all records must have a decision (i.e., include or exclude) any unresolved records at this stage may be lost and make it extremely difficult to track decisions later. Records are extracted in batches, with each reviewer receiving their own sets, maintaining familiarity from Stage 3.2. Each reviewer's files are saved in a dedicated shared folder, to support transparency and ease of access. Excluded records are also exported and kept for reference, providing an additional backup beyond the screening platforms archive. All exports should be systematically labelled for traceability (see Top Tip 2).

**Table:** 

Top Tip 3: Exclusion rationale
We recommend separating ‘exclusion category’ and ‘exclusion reason’ into two columns to provide clarity and flexibility during screening. Create drop‐down lists in Excel for the ‘exclusion category’ column can greatly simplify PRISMA flowchart preparation. Always include an ‘other’ category to allow emerging reasons to be captured and encourage reviewers to use optional notes columns to flag uncertainties for discussion.

#### 2.4.3 Full Text Screening

Full‐text screening is technically part of Stage 3: Study selection, but in practice, we found it more efficient to complete it at this stage once records have been extracted from the screening platform and full texts sourced. Whereas some screening platforms support full‐text screening, we found that a spreadsheet was easier to use as an educational tool. Reviewers independently screen their assigned records, documenting exclusion reasons (see Top Tip 3). A random 10% sample (see Top Tip 4) is double‐screened using a separate spreadsheet to maintain independent assessment.

#### 2.4.4 Extracting Data

The extraction process follows three phases:

1. Training and familiarisation: extraction tool collaboratively reviewed, the purpose of each column discussed to share understanding of the scope and aims of data collection
2. Pilot extraction: 10% of sources independently extracted to test effectiveness with amendments made to improve clarity or capture emerging areas of interest. Any changes are reflected in each reviewer's tool with pilot sources revisited for consistency.
3. Final extraction: Once refined, full extraction proceeds. As in Stage 4.3, 10% of sources are double‐extracted in a separate spreadsheet with conflicts resolved collaboratively. Differences in interpretation can provide valuable opportunities for reflexive discussion about how reviewers' disciplinary backgrounds and experiences shape interpretation. Where multiple interpretations remain plausible, these should be documented and considered during synthesis rather than prematurely collapsed into a single viewpoint.

**Table:** 

Top Tip 4: Random sample generation in excel
To generate a random sample in Excel, but one that will be easily reproducible when you are wanting to compare results, we sorted Columns A–Z by title and then picked a sample of 10% started with a letter of the teams choosing. These were then transferred into a new spreadsheet and the initial comments removed. Note the initial of the ‘checker’ in the box on the main spreadsheet.

### 2.5 Stage 5: Collating, Summarising and Reporting the Results

MAPS makes synthesis a collaborative interpretive process rather than locating it solely with the most experienced reviewer. The lead reviewer facilitates framework development, whereas junior reviewers draw on their immersion in the data to contribute to interpretation and learn how charted data become a written synthesis.

#### 2.5.1 Collaborative Construction and Reflexivity

Arksey and O'Malley described creating an analytic framework. In MAPS, this is pivotal to providing a structured yet inclusive way for those immersed in the data to integrate diverse perspectives. This step centres on a dedicated team meeting where reviewers discuss their findings and map an analytical structure guided by the research questions. Supporting Information S2 provides a worked example of this framework development process, illustrating how broad analytical domains can be translated into subcategories with space for contextual notes and reflexive observations. During discussions, team members often recognise perspectives they had not previously considered; these moments provide valuable insight into how their prior experiences shape interpretation. Reflexive details are captured in individual diaries and team notes.

*Illustrating how broad analytical domains can be translated into subcategories with space for contextual notes and reflexive observations*.

#### 2.5.2 Reporting the Results

The results are drafted, using the team's framework, by the lead reviewer, who does not extract data in MAPS and thus maintains a high‐level perspective supporting top‐level synthesis. The goal is to present findings and highlight gaps clearly, consistent with scoping review methodology. The wider team reviews the initial draft to confirm the synthesis accurately aligns with the collective interpretation. Reviewing the draft also allows junior reviewers to see how their extraction and collective interpretation have been represented in scholarly writing and to identify omissions or misinterpretations.

#### 2.5.3 Creating a Summary of Included Papers

This is standard practice in reporting but the use of colour‐coding throughout MAPS can streamline creation (see Top Tip 5), provided that colour choices account for accessibility, including colour‐vision differences. Sources from individual reviewers are combined, arranged alphabetically and systematically renamed (see Top Tip 2) after a master copy with original references is retained for traceability. The table contains top‐level metadata plus notes on the use of each source in the manuscript. An example completed summary table is provided in Supporting Information S1 (Table S3), which readers may adapt for their own reviews, supporting an accessible introduction to the evidence base in the results section.

**Table:** 

Top Tip 5: Colour coding
Use consistent colour coding across spreadsheets (e.g., green = included, yellow = discussion, red = excluded) to make screening intuitive and support later filtering (see Supporting Information S1 and S2). When undertaking the collaborative framework (Stage 5.1) assign each reviewer a distinct font colour and include a simple colour key to track contributions at a glance. Check accessibility needs and adapt the palette for colour‐vision differences, where possible. Colour filtering then enables rapid merging of included records into a single dataset for publication (Stage 5.3).

### 2.6 Optional Stage 6: Consultation Exercise

Arksey and O'Malley described stakeholder consultation as optional, but consistent with their rationale, and others [1], we strongly recommend its inclusion to enhance the relevance, applicability and impact of the review. Within MAPS, consultation also gives Junior Reviewers experience of how stakeholders interrogate, contextualise and extend review findings. This requires preparation and support from the lead reviewer, as well as planning and resourcing from the outset.

*Consultation also gives junior reviewers experience of how stakeholders interrogate, contextualise and extend review findings*.

## 3 Global Considerations

### 3.1 Software

Software was selected based on institutional support, accessibility and familiarity. Specific platforms can be chosen according to local resources and requirements, enabling adaptation of MAPS across different settings. Although dedicated screening and reference‐management software may improve efficiency, MAPS can be implemented using spreadsheet software alone, which provides a transparent record of decisions and is often familiar to students and educators. The examples in Table 2 are intended to illustrate available options rather than prescribe a specific approach.

**TABLE 2:** Software use in MAPS.

Software	Purpose in MAPS	Key features/notes
Rayyan (professional, web‐based)	Duplicate removal and independent title/abstract screening	Option to screen ‘blinded’ or ‘unblinded’, allowing teams to hide or reveal decisions as needed; tracks inclusion/exclusion and flags conflicts for discussion. Paid version used, though the free version is functional with sample‐size limits.
Zotero (v6.x, Windows/macOS)	Primary reference management, storage of full texts and metadata	Shared library supports team collaboration; browser extension enables quick capture of grey literature. Paid storage used, with a free version also available.
Microsoft Excel (Office 365)	Full‐text screening and data extraction	Structured spreadsheets support collaborative decision‐tracking and filtering across reviewers.

### 3.2 Support

Support is essential, particularly for novice‐reviewers. In our experience, undergraduate interns formed a peer‐support group via WhatsApp, whereas the lead reviewer remained accessible by email. Other messaging or communication platforms may be more appropriate depending on participants' location, access, institutional context and preferences. This blend of support helped maintain confidence and momentum throughout the review. Team meetings promoted development, collaboration and clear communication. Induction meetings prior to commencing the formal model are particularly important for setting expectations and establishing shared working practices.

*Support is essential, particularly for novice‐reviewers*.

## 4 Conclusion

Arksey and O'Malley provide the methodological architecture for scoping reviews. MAPS complements this by showing how the work can be organised within a mixed‐experience team. Defined roles, shared tools, staged calibration, structured feedback and collaborative interpretation allow substantive work to be distributed while maintaining methodological oversight and creating opportunities for research learning. Although these elements are not individually new, MAPS integrates them into a coherent process that teams can adapt to their own context. Future work will involve formally evaluating its utility and iteratively refining the framework as additional teams apply it, ensuring it remains responsive to evolving needs. We hope it provides a useful blueprint for teams undertaking collaborative scoping reviews.

## Author Contributions

**Heidi Stelling:** conceptualization, writing – original draft, writing – review and editing. **Megan Brown:** writing – review and editing. **Alex Boyle:** writing – review and editing. **Christina Higgins:** writing – review and editing. **Bryan Burford:** supervision, writing – review and editing. **Gillian Vance:** supervision, writing – review and editing.

## Funding

The authors have nothing to report.

## Ethics Statement

The authors have nothing to report.

## Conflicts of Interest

The authors declare no conflicts of interest.

## Supporting information


**Data S1:** Spreadsheet Examples: Examples of spreadsheets used to support different stages of the review process: (1) data extraction template; (2) extraction of additional sources; (3) summary of sources for publication; (4) tracking of backward and forward citations; and (5) targeted academic and grey literature searching.

**Data S2:** Collating Findings: Suggested framework to support the collaborative collation, organisation and synthesis of findings across the review team.

## Data Availability

Data sharing not applicable to this article as no datasets were generated or analysed during the current study.
