# Supplementary material for: The Multi‐Author Practical Scoping (MAPS) Model: Practical Guidance for Collaborative Scoping Reviews in Health Professions Education
Source: Clin Teach. 2026 Sep 14;23(5):e70523. doi: 10.1111/tct.70523 (PMC13574516; doi:10.1111/tct.70523)
Supplement: Supplementary file 1 [file TCT-23-e70523-s002.docx]

Collating Findings

## Aim

To systematically map and synthesise the international evidence on the factors that influence the recruitment and retention of midwives, in order to identify common drivers, barriers, and enabling strategies that affect maternity workforce sustainability.

## Research Questions

1. What are the key challenges and barriers that midwives face in entering and remaining in the profession?
2. What strategies or interventions have been implemented to support the recruitment and/or retention of midwives, and what is known about their effectiveness?
3. Do factors influencing midwifery workforce sustainability vary with context?

**Colour Key**

Christina, Alex, Heidi, Megan

# Recruitment

### Challenges/Barriers

| Domain | Reference | Comment |
| --- | --- | --- |
| Pre/During Training/Degree | | |
| Not Completing Degree | | |
| Lack of Psychological Support | C411-161 | BAME applicants felt disadvantaged by inadequate guidance and support through application procedures compared with peers with stronger networks or family connections in healthcare |
| Societal Expectations | A303-86 | A303-86: Conditions about not being pregnant or breastfeeding before or during training |
| Financial Issues | A303-67 | This was discussed in the meeting and more papers mentioned – is this complete? |
| Pre/During Degree | C411-1 | Training facilities lacked adequate resources, equipment, and teaching capacity, limiting learning opportunities and making the profession less attractive. Ongoing security issues in some areas deterred students from training or taking posts far from home, whilst for others it was culturally unacceptable to live away from family which reduced access to midwifery education |
| Educational pre-requisites | A303-86 | A303-86: country dependent but due to societal structure men were more likely to have the qualifications needed to enter profession, but unlikely to do due to societal norms |
| Perception of Midwifery | A303-266 | A303-266: Perception of midwifery being a difficult job with large responsibility |
| Not attracted to Profession | | |
| Lack of Role Models | C411-13, C411-58, C411-139, C411-161, C411-226 | BAME population felt lack of qualified role models, making entry seem less accessible/welcoming, C411-58: young students noted that peers/family members had little understanding of midwifery reflecting a barrier rooted in societal awareness of the profession. C411-139: lack of racially concordant role models which limited visibility of the profession for aspiring midwives of colour  Does this feature in retention too? |
| Cultural  Understanding | C411-58,  A303-282 | Resistance in places where the role of a MW was not traditionally trained – fear of new things/don’t see the need/not taking seriously/not respected; these may need to be multiple lines depending on references. Lack of trust; not welcomed into communities ?this may be more retention depending on how it is framed in the papers. C411-58: in some contexts, midwifery is perceived as lower-status compared with medicine which can discourage recruitment unless students have strong intrinsic motivations |
| Perception of the Role | A303-282,  A303-107 | A303-282: Perception of long difficult hours and shift work + fear of contact with blood/bodily fluids. A303-107: Not so much to do with not being attracted to the role, and more about being attracted to the role for the wrong reasons. Picking the profession due to thinking it is financially rewarding, not due to feeling like its the right profession because of care etc. Concerns that long term this will mean that |
| Structural Racism | C411-13, C411-139, C411-161 | BAME identified racism in admissions processes, biased assumptions from faculty, and microaggressions in training settings, which discouraged entry and progression. C411-139: experiences of systemic racism in admissions process, training environments and clinical placements all discouraging entry into midwifery |
| Post-Training/Degree Recruitment into Work | | |
| Cultural Norms | Reference missing? | Can’t work away from home as there is no suitable housing and culturally unacceptable to live alone. |
|  | C411-1 | Many faced external challenges such as violence and gender bias (gender norms and societal mistrust) that jeopardized their workforce participation |
| Organisational Fit | C411-23 | The way “organisational fit” is presented in recruitment materials can act as a barrier/enabler. Vague language and ambiguity in job adverts seem to deter applicants due to uncertainty about whether they align with organisational values. However, this also strengthened recruitment when values were clearly articulated |
| Lack of Preparedness During Transition | C411-188  A303-91 | Graduate MWs often entered employment processes e.g., interviews without adequate preparation which reduced confidence and limited success in securing roles. The uncertainty of transitioning from student to practitioner coupled with high-pressure interviews also contributed to stress and insecurity in entering the workforce  A303-91: ageing workforce creating situation where experienced veteran staff are retiring and newer qualified midwives don’t have the skills to replace them, and even when newer midwives are recruited they feel unable to deal with the workload so are more prone to burn out (links to retention too) |
| Structural Barriers | C411-226 | IEMs faced systemic barriers such as non-recognition of qualifications, culturally insensitive assessments (e.g., deemed to lack competence due to unfamiliarity with communication style) and limited bridging opportunities – few structured pathways exist to help IEMs adapt their knowledge and skills and their expertise often goes undervalued, which can discourage persistence in the profession |
| Lack of Infrastructure | A303-268 | Unwillingness to go and work in underserved areas (eg: rural/conflict affected areas) due to lack of local infrastructure (eg: medical equipment/supplies, |

**Any specific context examples or variations evident?**

**Gaps: area identified as lacking?**

EXTRACT ONLY….
